# An international qualitative study of ability and disability in ADHD using the WHO-ICF framework

**DOI:** 10.1007/s00787-017-0983-1

**Published:** 2017-03-28

**Authors:** Soheil Mahdi, Marisa Viljoen, Rafael Massuti, Melissa Selb, Omar Almodayfer, Sunil Karande, Petrus J. de Vries, Luis Rohde, Sven Bölte

**Affiliations:** 1 Department of Women’s and Children’s Health, Center of Neurodevelopmental Disorders (KIND), Paediatric Neuropsychiatry Unit, Karolinska Institutet, Stockholm, Sweden; 2 Center for Psychiatry Research, Stockholm County Council, Stockholm, Sweden; 3 Division of Child and Adolescent Psychiatry, University of Cape Town, Cape Town, South Africa; 4 ADHD Outpatient Program, Hospital de Clínicas de Porto Alegre, Federal University of Rio Grande do Sul, Porto Alegre, Brazil; 5 National Institute of Development Psychiatry for Children and Adolescents, São Paulo, Brazil; 6 ICF Research Branch, a cooperation partner within the WHO Collaborating Centre for the Family of International Classifications in Germany (at DIMDI), Nottwil, Switzerland; 7 Swiss Paraplegic Research, Nottwil, Switzerland; 8 Mental Health Department, KAMC-R, MNGHA, Riyadh, Saudi Arabia; 9 Learning Disability Clinic, Department of Paediatrics, Seth G.S. Medical College and K.E.M Hospital, Mumbai, India; 10 Child and Adolescent Psychiatry, Stockholm County Council, Stockholm, Sweden

**Keywords:** ADHD, Neurodevelopmental disorder, Impairment, Assessment, Psychiatry, ICD, DSM, Quality of life, Qualitative study

## Abstract

This is the third in a series of four cross-cultural empirical studies designed to develop International Classification of Functioning, Disability and Health (ICF, and Children and Youth version, ICF(-CY) Core Sets for Attention-Deficit Hyperactivity Disorder (ADHD). To explore the perspectives of individuals diagnosed with ADHD, self-advocates, immediate family members and professional caregivers on relevant areas of impairment and functional abilities typical for ADHD across the lifespan as operationalized by the ICF(-CY). A qualitative study using focus group discussions or semi-structured interviews of 76 participants, divided into 16 stakeholder groups. Participants from five countries (Brazil, India, Saudi Arabia, South Africa and Sweden) were included. A deductive qualitative content analysis was conducted to extract meaningful functioning and disability concepts from verbatim material. Extracted concepts were then linked to ICF(-CY) categories by independent researchers using a standardized linking procedure. In total, 82 ICF(-CY) categories were identified, of which 32 were related to activities and participation, 25 to environmental factors, 23 to body functions and 2 to body structures. Participants also provided opinions on experienced positive sides to ADHD. A high level of energy and drive, creativity, hyper-focus, agreeableness, empathy, and willingness to assist others were the most consistently reported strengths associated with ADHD. Stakeholder perspectives highlighted the need to appraise ADHD in a broader context, extending beyond diagnostic criteria into many areas of ability and disability as well as environmental facilitators and barriers. This qualitative study, along with three other studies (comprehensive scoping review, expert survey and clinical study), will provide the scientific basis to define ICF(-CY) Core Sets for ADHD, from which assessment tools can be derived for use in clinical and research setting, as well as in health care administration.

**Electronic supplementary material:**

The online version of this article (doi:10.1007/s00787-017-0983-1) contains supplementary material, which is available to authorized users.

## Background

Attention-Deficit Hyperactivity Disorder (ADHD) is a neurodevelopmental disorder, affecting 3–7% of children and adults worldwide [1–4]. Besides the core behavioural symptoms of inattention, hyperactivity and impulsivity [5], the definition of ADHD includes related interference with social, educational or occupational functioning, and an associated reduced quality of life across the lifespan [6–10]. Neurodevelopmental and psychiatric comorbidity is common in ADHD [11], further contributing to functional impairment [12]. Interestingly, ADHD has also been reported to include specific strengths, such as creativity, hyper-focusing and high levels of energy [13], although these have not been consistently supported by other research findings [14, 15]. While ADHD typically causes impairment across different life domains, the degree and profile of its individual impact might differ significantly. Therefore, the availability of internationally accepted, standardized classification tools for individual assessment of functional ability and disability in those living with ADHD may be helpful in clinical, research and health care administration settings.

In 2001, the World Health Organization (WHO) released the International Classification of Functioning, Disability and Health (ICF) with the aim to provide a comprehensive and universal framework to describe different aspects of functioning and disability for all health-related conditions [16]. In 2007, the Children and Youth version of the ICF, the ICF(-CY), was published, designed specifically to capture the functional abilities and disabilities in developing individuals, by adding and expanding on the categories of existing ICF categories [17]. The ICF(-CY) was designed to complement the International Classification of Diseases-Tenth Version (ICD-10), which defines and describes health conditions, symptoms, complaints, and where possible, causes of injury or diseases [18]. The ICF(-CY) is based on a bio-psycho-social model of functioning, which conceptualizes disability and ability as the result of an interaction between a health condition with individual physical and personal characteristics, and environmental factors. The ICF(-CY) provides detailed classifications of functioning and disability in the areas of body functions (i.e. physiological functions of body systems), body structures (i.e. anatomical parts of the body), activities (i.e. execution of tasks), participation (i.e. involvement in life situations), and environment (i.e. physical, social and attitudinal environment) [16]. For each of these components, aspects of functioning can be described at different levels of depths. The first level includes chapters giving an overview of the areas of functioning covered by the nomenclature. These chapters in turn comprise information about specific categories of functioning that are hierarchically structured with up to three level of increasing detail, as demonstrated by the following example from the activity and participation component:

- Level 1 chapter: d5 self-care
- Level 2 category: d570 looking after one’s health
- Level 3 category: d5702 maintaining one’s health
- Level 4 category: d57022 avoiding risks of abuse of drugs or alcohol

Personal factors, such as gender, race, educational level, coping styles, are also deemed highly important and are included in the framework of the ICF(-CY). However, given their significant social and cultural variability, they have not been classified in the ICF(-CY).

The ICF(-CY), which includes all ICF categories plus additional ones for youth, consists of 1685 categories (Body functions, *k* *=* 531; Body structures, *k* *=* 329; Activities and participation, *k* *=* 552; and Environmental factors, *k* *=* 273). They serve to fine-map functioning and disability in all health conditions in research, clinical and healthcare administration settings for diagnostic, treatment, documentation and reimbursement purposes [19, 20]. However, even though the comprehensiveness of the ICF(-CY) is an advantage, to use all its categories to describe a specific health condition is both unnecessary and impractical, as many categories may be irrelevant to specific disorders. To address this issue, the development of ICF(-CY) Core Sets was initiated, which involves a rigorous and systematic scientific approach to select ICF(-CY) categories that are most relevant to individuals with a particular health condition. The development of Core Sets comprises a qualitative study (current study), a literature review (“research perspective”), an expert survey (“expert perspective”) and a clinical study (“clinical perspective”). Each study aims to capture general and unique features of functioning and disability related to a specific health condition, ensuring that the process includes a diverse range of professionals and stakeholders across all of the six WHO regions. Therefore, the current study is part of a larger systematic effort that will conclude with the creation of standardized ICF(-CY) Core Sets for ADHD. ICF(-CY) Core Sets for Autism Spectrum Disorder (ASD) are also being developed as part of this project with the results reported in separate publications [21–23]. A general description of the ADHD ICF(-CY) Core Sets development process has been published in a previous issue of this journal [24].

The objective of this study was to capture the perspectives pertaining to ADHD, as expressed by stakeholders from various WHO-regions, and link them to ICF(-CY) categories. To facilitate comparison with our other ADHD Core Set preparatory studies [13, 35], an exploratory secondary objective was added to determine the consistency of identified ICF(-CY) concepts. For this purpose, a qualitative and mixed methodology study as outlined by the WHO [25] was conducted. It involved focus group discussions and individual semi-structured interviews with participants across ages diagnosed with ADHD, self-advocates, immediate family members and professional caregivers, regarding functional disability and ability characteristics of ADHD, as well as facilitators and barriers to functioning. Together with the other preparatory research mentioned above, this study will provide the basis for an international ICF(-CY) Core Sets consensus conference, during which a group of independent ADHD experts, representing different professions and all WHO-regions, will follow a formal decision-making process on which ICF(-CY) categories to be included in the ICF(-CY) Core Sets for ADHD.

## Methods

### Design and procedure

**Fig. 1:**
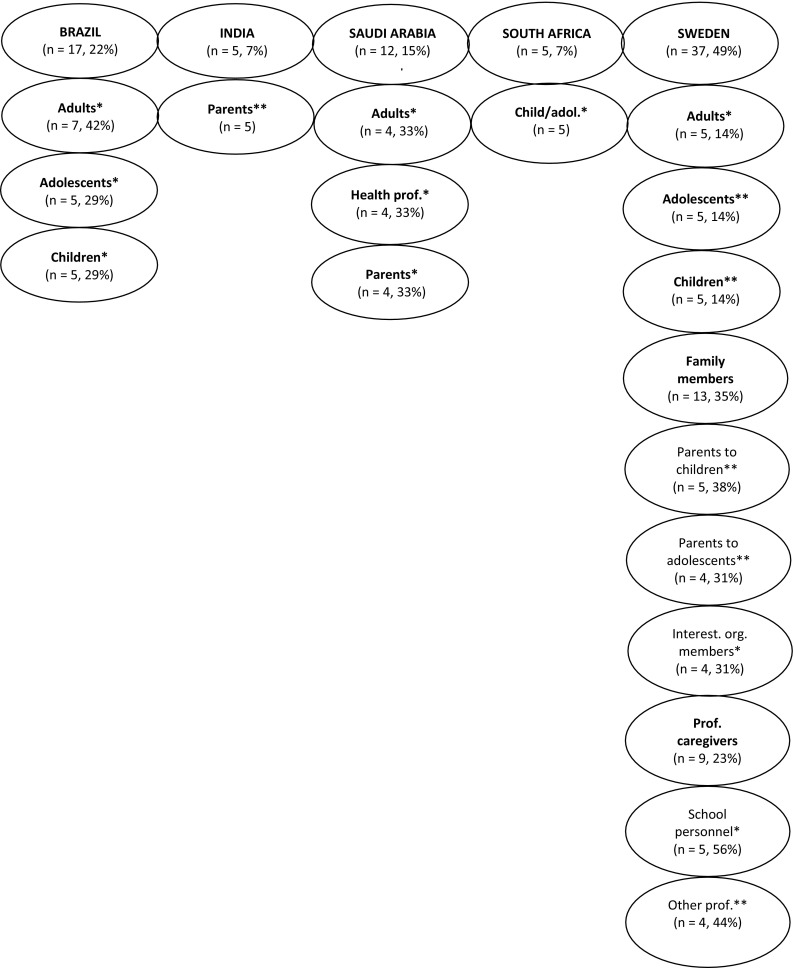
Composition of stakeholder groups by country. *Asterisk* focus group discussions, *double asterisk* individual semi-structured interviews

The study was approved by the regional ethical review board in Stockholm and by local ethics review boards in participating countries. Written and verbal informed consent was obtained from each participant and/or parent or legal guardian prior to participation. A qualitative methodology, combining focus group discussions and individual semi-structured interviews, was used for data collection. To achieve a broad representation, 76 participants were divided into 16 groups (Fig. 1) according to age (child, adolescent, adult), perspective (own diagnosis, family members, professional, interest organization) and WHO country (region), namely Brazil (The Americas), India (South-East Asia), Saudi Arabia (Eastern Mediterranean), South Africa (Africa) and Sweden (Europe). In previous preparatory qualitative studies of ICF Core Sets [26–29], four to six focus group discussions were required to achieve data saturation. This study included 10 focus groups plus additional 28 individual interviews. The substantial contributions of international focus groups were made for two reasons: first, to meet the requirements for including a global and cross-cultural perspective on functioning and disability pertinent to ADHD; second, to enable future novel hypotheses of cross-cultural effects on neurodevelopmental disorders, such as ADHD, as these have shown to affect treatment and assessment of ADHD [30]. For these reasons, it was deemed necessary to include a larger sample of stakeholder groups across different WHO-regions. The group size of focused discussions is usually based on topic complexity, with six to ten participants being optimal [31]. However, for the focus groups in present study, we anticipated a range of potential ADHD-related difficulties (e.g. impulsiveness, organizational skills) that might interfere with focus group conductance. For this reason, smaller groups of four to eight participants were deemed more appropriate to facilitate communication between group members, and to ensure high-quality data collection. Individual semi-structured interviews were conducted to accommodate for logistical challenges (e.g. last minute cancellations of focus group participants or expressed preferences of several participants to take part in more intimate and anonymous interviewing). The group discussions generally lasted between 60 and 120 min (including short breaks), while the individual interviews typically took 15–60 min to complete. Focus group discussions and individual interviews were led by a moderator, either a clinician or clinical researcher experienced in ADHD. All group discussions and individual interviews were audio-recorded, with the exception of the stakeholder group in India, where the participants did not consent to having their interviews retained. Group discussions and individual interviews were transcribed verbatim and then translated into English by approved translators.

### Participants

In total, 82 individuals who fulfilled the criteria for study participation were contacted between February and December 2015. Inclusion criteria for participants were either (1) having a well-documented primary clinical diagnosis of ADHD combined presentation or an ADHD subtype presentation (predominantly inattentive or hyperactive-impulsive) according to the diagnostic criteria of the ICD-10, DSM-IV/-TR or DSM-5 and/or receiving treatment for ADHD, (2) being an immediate family member, professional caregiver, or other closely involved in the everyday life of an individual fulfilling criteria (1). Participants were excluded from the study if they were younger than 7 years of age or could not communicate in the language of the country they resided in. Recruitment of participants was made via clinical research teams in each country, and via invitations in collaboration with local and national interest organizations for ADHD. In 10 stakeholder groups, data were collected through focus group discussions and the rest by employing individual-semi structured interviews. The contributions of stakeholder groups in respective countries were made by members of the project Steering Committee (see acknowledgement), a group of ADHD experts from all WHO-regions, who provide support and guidance on the project.

### Material

For the group discussions and individual interviews, seven general items covering all WHO ICF(-CY) components (6 items) as well as one question on strengths associated with ADHD were included in the interview guide (see Online Appendix 1). The six items that covered all WHO ICF(-CY) components were predetermined, following the study protocol developed by the WHO and ICF Research Branch [25]. To clarify these items, illustrative examples or (probing) questions were added and adapted to the respective group or individual, for instance age, diagnostic status, level of functioning, professional group, etc. Prior to the discussions and interviews, the participants received a case record form (CRF), which they were asked to complete and return before or at the focus group discussions or individual interviews. Participants with ADHD received a CRF that captured information related to date of birth, gender, diagnosis (presentation), psychiatric and somatic comorbidity, marital status, living situation, formal education, work status and current treatments. Other participants (immediate family members and professional caregivers) received a CRF that captured information related to their own socio-demographic factors (i.e. gender, age). Moreover, the CRF inquired about the subtype of ADHD diagnosis and age group of those individuals they were related to, or worked with.

### Analysis of verbal material: meaningful concepts and ICF(-CY) linking

Researchers carefully examined the verbatim transcripts from the focus group discussions and individual semi-structured interviews. A deductive qualitative content analysis [32] was performed to first extract meaningful units from the verbatim transcripts. A meaningful unit within the ICF(-CY) Core Set preparatory research does not follow linguistic grammatical rules, rather the text is divided where a shift in meaning is observed. Thereafter, based on the meaningful units, the researchers extracted meaningful concepts that were pertinent to functioning. “Meaningful concepts” refer in this context to concepts that reflect the essence of what statements are saying. For example, the meaningful concepts of the statement “I don’t have energy enough to do my housework” are “lack of energy” and “do housework”. Identified meaningful concepts were linked to ICF(-CY) categories following a set of formal rules and procedures as determined by the ICF Research Branch [33], a cooperation partner within the WHO Collaborating Center for the Family of International Classifications in Germany (at DIMDI). The linking rules and procedures provided information on how to link the concepts to ICF(-CY) categories, as well as what to do in cases where linkage is not possible. These cases include (1) personal factors, if the concept is not contained in the ICF(-CY), but is clearly a personal factor as defined in the ICF-(CY); (2) not covered, if the concept is not contained in the ICF(-CY) and also is not a personal factor; (3) non definable, if the information provided in the concept is not sufficient for assigning it to a specific ICF-(CY) category; and (4) health condition, if the concept refers to a diagnosis or health condition. ADHD-related strengths that were mentioned by the stakeholders were also analysed and linked to ICF(-CY) categories as stated above. Strengths that could not be linked to ICF(-CY) categories were summarized as recurring themes.

To ensure the consistency of linking results for each focus group and semi-structured interview, both the identification of meaningful concepts and linking of ICF(-CY) categories were conducted by two independent researchers. To capture different cultural expressions in the participants’ answers, at least one independent researcher was included from the countries that were involved in this study (excluding India, as only one stakeholder group was conducted there). In total, seven independent researchers (AF, HA, JH, MV, NA, RM, SM) were involved in the linkage. To prepare for the linking of actual data from the focus groups and individual interviews, the researchers received linking exercises that were provided by the ICF Research Branch (http://www.icf-research-branch.org). Linking results were compared and consensus discussions were used to resolve disagreements. In situations where consensus could not be reached, the coordinator at ICF Research Branch (MS) was available to make the final decision. However, this option was never used, as all disagreements were resolved by discussion. To investigate possible differences in inter-rater agreement between the two methods used for data collection (group discussions versus semi-structured interviews), Cohen’s Kappa was calculated separately. Kappa coefficients for the second level ICF(-CY) categories in the focus group discussions and semi-structured interviews were, respectively, *ĸ* = 0.68 (SE = 0.01) with a confidence interval of *ĸ* = 0.66–0.70, and *ĸ* = 0.70 (SE = 0.02) with a confidence interval of *ĸ* = 0.67–0.73. These indicate substantial agreement, irrespective of data collection method applied.

### Consistency of quoted ICF(-CY) categories

Meaningful functioning concepts that were identified in the responses to the seven items employed in the group discussions and individual interviews were extracted and linked to ICF(-CY) codes. To examine the consistency of quoted ICF(-CY) categories, orienting frequency analyses were conducted on the transcriptions across the different stakeholder groups. In this study, ICF-(CY) categories are presented at the second level. If a concept is linked to a third- or fourth-level ICF(-CY) category, the corresponding second-level category is reported. To avoid favouring participants that repeatedly expressed similar statements or were prompted by other participants’ responses, an ICF(-CY) category was only counted once for each stakeholder group that involved focus group discussion (max. 10) or individual semi-structured interviews (max. 6). Even though the possibility of prompting did not exist for those stakeholders who took part in individual semi-structured interviews, the same rule of counting was applied here to avoid favouring the responses of interviewed participants. Consistent with previous preparatory ICF Core Sets qualitative studies [26, 27], to fully exploit the verbal material and to maximize sensitivity of all perspectives put forward by stakeholders, an ICF(-CY) category that was mentioned at least once in any group was included in the list of candidate categories. For ADHD-related strengths, only the consistency of recurring themes was summarized to facilitate comparison with our other ADHD Core Set preparatory study, namely the expert survey [13].

## Results

### Sample

Of the 82 individuals eligible for study participation, 76 completed the group discussions or individual interviews. Attrition in 6 participants was due to not showing up for group discussions (*n* *=* 2) or late decline to participate in the study (*n* *=* 1). In addition, three children with ADHD were initially included in the study, but could not complete the focus group discussion due to restlessness. Table 1 summarizes the stakeholder groups, gender composition and age of participants who were included in the final analysis. Among the individuals diagnosed with ADHD, combined ADHD was the most frequent presentation (*n* *=* 17) along with the inattentive presentation (*n* *=* 12), followed by the hyperactive-impulsive presentation (*n* *=* 8) and unspecified ADHD (*n* *=* 2). Two participants chose to not respond to the question. Most immediate family members stated that their relative was diagnosed with the combined presentation (*n* *=* 12), followed by an inattentive presentation (*n* *=* 5). Only one family member indicated that their relative with ADHD was diagnosed with the predominantly hyperactive/impulsive presentation. Members of the interest organization mentioned they were in contact with individuals from the entire spectrum of ADHD (*n* *=* 4). Most commonly reported treatment methods by those diagnosed with ADHD were pharmacotherapy (*n* *=* 16), followed by pharmacotherapy and psychosocial treatment combined (*n* *=* 12). Two participants with ADHD received psychosocial treatment only, while eight did not report any current treatment for ADHD. The remaining three participants with ADHD did not respond to the question. Participating individuals diagnosed with ADHD lived with their parents (*n* *=* 3), independently (*n* *=* 3), with a partner (*n* *=* 3), or in communal settings (*n* *=* 3). The rest did not respond to the question (*n* *=* 2). Thirteen diagnosed individuals with ADHD reported to have university or college studies as their highest level of education, whereas twenty-six indicated high school or primary school studies as their highest level of education. Only one participant came from another level of education, namely vocational education, and one did not respond to the question. Individuals with ADHD were students (*n* *=* 25), full-time employed (*n* *=* 6), part-time employed (*n* *=* 2), self-employed (*n* *=* 2), engaged in volunteer work (*n* *=* 2), or held different forms of work and employment (*n* *=* 2), while one was on sick leave. Nine of those diagnosed with ADHD reported having at least one additional diagnosis.

**Table 1:** Characteristics of study participants

Stakeholder groups	Size of group *N* (%)	Gender (male) *N* (%)	Age *M* (SD)	Age range
Clients	41 (54)	25 (61)	21 (12.9)	7–61
Children	13 (17)	9 (69)	10 (1.6)	7–12
Adolescents	12 (16)	8 (67)	15 (1.3)	13–17
Adults	16 (21)	8 (50)	35 (10.1)	24–61
Immediate family members	22 (29)	4 (18)	45 (8.9)	31–58
Parents	18 (24)	4 (22)	46 (9.2)	31–58
Interest organization members^a^	4 (5)	0	40 (5.7)	35–47
Professional caregivers	13 (17)	4 (31)	42 (9.8)	30–59
School personnel^b^	5 (7)	1 (20)	49 (8.4)	40–59
Other professionals^c^	8 (10)	3 (37)	36 (6.4)	30–47

^a^Interest organization members consisted of individuals who had family relatives diagnosed with ADHD. The members work with raising awareness about ADHD and support those who have the diagnosis, as well as their relatives

^b^School personnel included teachers, special educators and principals

^c^Other professionals included health professionals (e.g. psychiatrists, psychologists, etc.) and individuals who work closely with individuals with ADHD in daily life, such as personal assistants and residential caregivers

### Meaningful concepts and ICF(-CY) linking

The analysis of the 16 groups yielded a total of 3021 meaningful concepts related to functioning. These concepts could be linked to 82 second-level ICF(-CY) categories, 243 personal factors (e.g. self-esteem, creativity, sense of humor), 152 non-definable codes (e.g. structure, understanding, body problems), 120 not covered codes (e.g. bullying, education programs for parents, crime), and 17 health condition codes (e.g. dyslexia, autism, anxiety). Meaningful concepts that were linked to third or fourth level ICF(-CY) categories were aggregated to second-level categories. Different meaningful concepts that expressed similar functions were linked to the same ICF(-CY) category. For example, difficulties in relating with peers and maintaining friendships are two separate meaningful concepts, but they were linked to the same aggregated second-level ICF(-CY) category, namely d750 informal social relationships. When performing data saturation [34], only one second-level ICF(-CY) category was found to be missing out of the 82 if data was added from study sites outside of Sweden. If the sample only contained responses from diagnosed individuals, 71 second-level categories would have been covered. An additional ICF(-CY) category would not have been identified if stakeholders only included families and diagnosed individuals.

Categories were found in each of the four ICF-(CY) components: activities and participation (*k* *=* 32), environmental factors (*k* *=* 25), body functions (*k* *=* 23) and body structures (*k* *=* 2). Table 2 shows the second-level categories that were identified in the activities and participation component and their consistency across stakeholder groups. Identified categories are spread across all of the nine chapters of this component, i.e. d5 self-care (e.g. caring for body parts and dressing, *k* *=* 6), d1 learning and applying knowledge (e.g. focusing and directing attention, *k* *=* 5), d2 general demands and tasks (e.g. undertaking a single task and carrying out daily routine, *k* *=* 5), d7 interpersonal relationships and interactions (e.g. complex interpersonal interactions and informal social relationships, *k* *=* 5), d6 domestic life (e.g. doing housework and assisting others, *k* *=* 4), d4 (e.g. fine hand use and moving around, *k* *=* 3), d8 major life areas (e.g. school education and engagement in play, *k* *=* 2), d3 communication (e.g. receiving spoken messages, *k* *=* 1) and d9 community, social and civic life (e.g. recreation and leisure, *k* *=* 1).

**Table 2:** Identified ICF(-CY) categories from the activities and participation component and consistency across stakeholder groups

Second-level ICF(-CY) category	Chapter level ICF(-CY) category	*N*
d160 Focusing attention	d1 Learning and applying knowledge	7
d161 Directing attention	d1 Learning and applying knowledge	7
d172 Calculating	d1 Learning and applying knowledge	5
d175 Solving problems	d1 Learning and applying knowledge	4
d177 Making decisions	d1 Learning and applying knowledge	4
d210 Undertaking a single task	d2 General tasks and demands	12
d220 Undertaking multiple tasks	d2 General tasks and demands	6
d230 Carrying out daily routine	d2 General tasks and demands	10
d240 Handling stress and other psychological demands	d2 General tasks and demands	9
d250 Managing one’s own behaviour	d2 General tasks and demands	11
d310 Communicating with—receiving—spoken messages	d3 Communication	3
d440 Fine hand use	d4 Mobility	5
d455 Moving around	d4 Mobility	6
d470 Using transportation	d4 Mobility	5
d510 Washing oneself	d5 Self-care	4
d520 Caring for body parts	d5 Self-care	6
d530 Toileting	d5 Self-care	3
d540 Dressing	d5 Self-care	5
d570 Looking after one’s health	d5 Self-care	8
d571 Looking after one’s safety	d5 Self-care	4
d630 Preparing meals	d6 Domestic life	3
d640 Doing housework	d6 Domestic life	6
d650 Caring for household objects	d6 Domestic life	5
d660 Assisting others	d6 Domestic life	6
d710 Basic interpersonal interactions	d7 Interpersonal interactions and relationships	5
d720 Complex interpersonal interactions	d7 Interpersonal interactions and relationships	13
d740 Formal relationships	d7 Interpersonal interactions and relationships	4
d750 Informal social relationships	d7 Interpersonal interactions and relationships	9
d760 Family relationships	d7 Interpersonal interactions and relationships	6
d820 School education	d8 Major life areas	12
d880 Engagement in play	d8 Major life areas	3
d920 Recreation and leisure	d9 Community, social and civic life	13

*N* number of stakeholder groups that mentioned the ICF(-CY) category

Second-level categories identified in the environmental factors component and their consistency across stakeholder groups are presented in Table 3. Categories in this component included all its five chapters, i.e. e4 attitudes (e.g. attitudes of immediate family members and other professionals, *k* *=* 8), e3 support and relationships (e.g. support from immediate family members and other professionals, *k* *=* 7), e1 products and technology (e.g. medication and cell-phones, *k* *=* 5), e5 Services, systems and policies (e.g. education and health services, *k* *=* 4) and e2 natural environment and human-made changes in environment (e.g. sound, *k* *=* 1).

**Table 3:** Identified ICF(-CY) categories from the environmental factors component and consistency across stakeholder groups

Second-level ICF(-CY) category	Chapter level ICF(-CY) category	*N*
e110 Products or substances for personal consumption	e1 Products and technology	14
e115 Products and technology for personal use in daily living	e1 Products and technology	12
e125 Products and technology for communication	e1 Products and technology	8
e130 Products and technology for education	e1 Products and technology	4
e140 Products and technology for culture, recreation and sport	e1 Products and technology	2
e250 Sound	e2 Natural environment and human-made changes to environment	7
e310 Immediate family	e3 Support and relationships	14
e315 Extended family	e3 Support and relationships	3
e320 Friends	e3 Support and relationships	9
e325 Acquaintances, peers, colleagues, neighbours and community members	e3 Support and relationships	10
e330 People in positions of authority	e3 Support and relationships	6
e340 Personal care providers and personal assistants	e3 Support and relationships	7
e360 Other professionals	e3 Support and relationships	10
e410 Individual attitudes of immediate family members	e4 Attitudes	8
e415 Individual attitudes of extended family members	e4 Attitudes	3
e420 Individual attitudes of friends	e4 Attitudes	5
e425 Individual attitudes of acquaintances, peers, colleagues, neighbours and community members	e4 Attitudes	7
e430 Individual attitudes of people in positions of authority	e4 Attitudes	7
e440 Individual attitudes of personal care providers and personal assistants	e4 Attitudes	4
e455 Individual attitudes of other professionals	e4 Attitudes	6
e460 Societal attitudes	e4 Attitudes	7
e580 Health services, systems and policies	e5 Services, systems and policies	4
e585 Education and training services, systems and policies	e5 Services, systems and policies	10
e590 Labour and employment services, systems and policies	e5 Services, systems and policies	4
e595 Political services, systems and policies	e5 Services, systems and policies	3

*N* number of stakeholder groups that mentioned the ICF(-CY) category

Table 4 shows the second-level categories that were identified in the body functions component and their consistency across stakeholder groups. The majority of categories here concerned chapter b1 mental functions (e.g. psychomotor control, energy and drive functions, attention; *k* *=* 16). The other categories were identified in chapters b4 functions of the cardiovascular, haematological, immunological and respiratory systems (e.g. heart functions and exercise tolerance functions; *k* *=* 2), b5 functions of the digestive, metabolic and endocrine systems (e.g. weight maintenance functions and sensations associated with the digestive system; *k* *=* 2), b7 neuromusculoskeletal- and movement-related functions (e.g. coordination, clumsiness; *k* *=* 2) and b2 sensory functions and pain (e.g. sensation of pain; *k* *=* 1).

**Table 4:** Identified ICF(-CY) categories from the body functions component and consistency across stakeholder groups

Second-level ICF(-CY) category	Chapter level ICF(-CY) category	*N*
b114 Orientation functions	b1 Mental functions	3
b117 Intellectual functions	b1 Mental functions	5
b122 Global psychosocial functions	b1 Mental functions	4
b125 Dispositions and intra-personal functions	b1 Mental functions	6
b126 Temperament and personality functions	b1 Mental functions	15
b130 Energy and drive functions	b1 Mental functions	14
b134 Sleep functions	b1 Mental functions	9
b140 Attention functions	b1 Mental functions	16
b144 Memory functions	b1 Mental functions	15
b147 Psychomotor functions	b1 Mental functions	16
b152 Emotional functions	b1 Mental functions	14
b156 Perceptual functions	b1 Mental functions	5
b160 Thought functions	b1 Mental functions	10
b164 Higher-level cognitive functions	b1 Mental functions	12
b167 Mental functions of language	b1 Mental functions	3
b180 Experience of self and time functions	b1 Mental functions	7
b280 Sensation of pain	b2 Sensory functions and pain	11
b410 Heart functions	b4 Functions of the cardiovascular, haematological, immunological and respiratory systems	4
b455 Exercise tolerance functions	b4 Functions of the cardiovascular, haematological, immunological and respiratory systems	4
b530 Weight maintenance functions	b5 Functions of the digestive, metabolic and endocrine systems	4
b535 Sensations associated with the digestive system	b5 Functions of the digestive, metabolic and endocrine systems	3
b760 Control of voluntary movement functions	b7 Neuromusculoskeletal and movement-related functions	10
b765 Involuntary movement functions	b7 Neuromusculoskeletal and movement-related functions	3

*N* number of stakeholder groups that mentioned the ICF(-CY) category

The two second-level categories identified in the body structures component came from s1 structures of the nervous system (structures of brain, *n* *=* 5) and s7 structures related to movement (structure of head and neck region, *n* *=* 5) (Table 5).

**Table 5:** Identified ICF(-CY) categories in the body structures component and consistency across stakeholder groups

Second-level ICF(-CY) category	Chapter level ICF(-CY) category	*N*
s110 Structure of brain	s1 Structures of the nervous system	5
s710 Structure of head and neck region	s7 Structures related to movement	5

*N* number of stakeholder groups that mentioned the ICF(-CY) category

### ADHD-related strengths

The majority of the participants (*n* *=* 54, 71%) indicated positive sides to ADHD and named one or more strengths related to the condition. Only 7 (9%) did not report any positive side, and 15 (20%) felt unable to respond to the question. Out of the 15 participants who were unable to respond to the question, 13 (87%) of them were individuals diagnosed with ADHD, mostly children (*n* *=* 6, 46%). Of those who reported no positive sides to ADHD, the large majority were immediate family members (*n* *=* 5, 71%). Strengths reported included b130 energy and drive functions, which suggested to make it easier to engage in physical exercises (e.g. swimming, football) and to achieve personal goals and face general demands and challenges in life (e.g. study before exams, meet deadline dates for work-related tasks). Other examples included creativity, enabling to “think outside of the box”. Furthermore, d161 directing attention (hyper-focus) was mentioned as strength, provided that the activity or topic was a core interest of the individual. Specific positive attributes in b126 temperament and personality functions (i.e. agreeableness and willingness to assist others) were also recurrently mentioned. Table 6 summarizes the most recurrent strengths identified in this study and their consistency across stakeholder groups.

**Table 6:** Absolute frequencies of recurring ADHD-related abilities and strengths

ADHD-related abilities and strengths	*N* (%)
b130 Energy and drive functions	11
Creativity	7
b126 Temperament and personality functions	5
d161 Directing attention	5

## Discussion

In preparation for official WHO ICF(-CY) Core Sets for ADHD, the current international qualitative study aimed to investigate the experiences and perspectives of individuals with ADHD, self-advocates, immediate family members and professional caregivers on disability and abilities pertinent to ADHD, as well as facilitators and barriers to functioning. Categories were identified in all four ICF(-CY) components, mainly from activities and participation, but also several environmental factors and body functions. Very few body structures were considered to be relevant. The activities and participation component and environmental factors were described comprehensively, as evidenced by the fact that categories were covered in all nine, respectively, five chapters. In the body functions component, many different aspects of mental functions were considered to be important. Additionally, our study identified evidence of strengths associated with ADHD, such as high level of energy, creativity, hyper-focus, agreeableness, and willingness to assist others.

The large number of ICF-(CY) categories identified across all of the components supports the notion that ADHD impacts on broad areas of body functioning and everyday life adaptive skills. Nearly 70% of all body functions categories covered in this study were from the b1 mental functions chapter, which is consistent with ADHD being conceptualized as neurodevelopmental and behavioural disorder. A total of 16 different mental functions were identified in this study, which demonstrates that cognitive functions are deemed crucial in ADHD. Our study also revealed physical alterations (e.g. body coordination) and sensory issues (e.g. sensation of pain) to be related to ADHD. The impact of ADHD on everyday life was described comprehensively. Consistent with previous studies on ADHD and social functioning [8, 36], this study identified five aspects of social interactions and relationships to be affected by ADHD. Examples include family relationships and informal social relationships, such as creating and maintaining interactions with friends and peers. Furthermore, ADHD was described to impact formal relationships, such as relating with persons in authority. Engagement in recreation and leisure activities, including participating in social events, were captured in this study as well. In line with previous research on ADHD and its impact on academic achievement [6], school disabilities were identified in this study. Other activities related to school, such as undertaking tasks and maintaining attention on homework assignments, were mentioned to be affected by ADHD too. Although occupational functioning was not covered in this study, some participants described having supportive individuals in the work environment that helped them with their tasks. These include people in positions of authority (e.g. employers) and colleagues. Their attitudes towards individuals with ADHD were also described to be positive, which made it easier to meet deadline dates and complete required work assignments. The categories in the environmental factors component can either functionally be perceived as a barrier or facilitator by the individual. For example, medication (i.e. e110 products or substances for personal consumption) might be experienced both in terms of relief from core symptoms of ADHD, but also generating functional challenges (e.g. sleep problems) owing to side effects. Compared to the other ICF(-CY) components, body structures were less commonly identified. Structure of brain was pointed-out as correlate of the many mental functions that were reported in this study. Interestingly, although not formally linkable to ICF(-CY) categories, a large number of personal factors was identified in this study, indicating that individual personal characteristics and resources are pivotal for handling of ADHD. The latter data combined with the results from the other three preparatory studies [13, 24, 35] on personal factors provide an additional valuable future option to analyse the data set in terms of overarching personal factors relevant to the management of ADHD.

Contrary to our other preparatory studies, published in previous issues of this journal [13, 35], this qualitative study did not capture any aspect related to occupational functioning. The current study did, however, identify a wider range of environmental factors relevant to ADHD, compared to the earlier ones. This suggests that environmental factors are considered more important for functioning in ADHD by subjects diagnosed with the condition, their immediate family members and professional caregivers than the current research literature indicates and ADHD experts suggest. Recurring themes that were identified in this study, such as attention, psychomotor functions, recreation and leisure, complex interpersonal interactions and immediate family, were also found to be relevant in the literature review [35] and expert survey [13].

This study is amongst the first to explore specific strengths in ADHD from an international and first hand perspective. Overall, the opinions stated were rather broad and not straightforward. In addition, several participants felt unable to mention any positive aspects related to ADHD, even when explicitly prompted. The most recurring themes about positive sides were creativity, high energy level, hyper-focus and sympathetic personality traits such as agreeableness and empathy. Still, there is currently little or no empirical support for such strengths outside of this study [14, 15]. However, importantly, these positive aspects were also identified in our earlier international expert survey, which included 174 experienced ADHD scientists and clinicians from 11 different professional backgrounds and 45 countries [13]. Thus, in combination with results from the other preparatory studies, well-grounded novel hypotheses for future research can be generated within this area of topic.

The current study presents with some methodological challenges. The generalizability of the consistency of recurring ICF(-CY) categories across groups might be questioned, as the analyses were based on uneven sample sizes and compositions, i.e. half of the stakeholder groups came from Sweden and not all were equally represented. However, these orienting frequency analyses only reflect the consistency of ICF(-CY) categories across groups and were mainly conducted to facilitate comparisons with the other two previous preparatory studies [13, 35]. The primary aim of this study was to capture the experiences of health-related functioning in ADHD by involving a diverse range of stakeholders and WHO-regions, and our saturation analyses showed that identified categories were probably quite exhaustive for ADHD in general. The involvement of several culturally diverse countries also generated challenges concerning transcriptions to English. Proper translation of specific cultural expressions and their exact connotation can be difficult or even impossible. In cases where an English equivalent was missing, similar terms were used. While the linking was conducted in collaboration with researcher located at one center for reasons of standardization and practicability, future studies might consider using independent researchers doing directly the linking of ICF(-CY) categories in their native language. Another possible weakness of this study is the non-involvement of business colleagues or employers of individuals with ADHD in the focus group discussions and individual interviews. Work is an important arena for individuals with ADHD and some functional abilities and disabilities might only be observable in work settings and perceived by colleagues or employers. However, there are given ethical and practical challenges to involve these groups in research. One challenge the current study faced was to adapt the focus group and interview items to the different age groups of individuals with ADHD. Some children and young adolescents found the questions rather difficult to discuss. Examples were given to clarify each of the questions, but in some cases it was still difficult for the younger participants to respond.

Despite the limitations, this study managed to identify a wide range of functional abilities and disabilities in individuals with ADHD by involving multiple stakeholders and WHO-regions. Compared to previous ICF qualitative studies [27–29], this study had a very heterogeneous and international sample of participants. The involvement of primary informants, namely diagnosed individuals, provides researchers with unique insights into how ADHD impacts various areas of daily life functioning. It offers, more importantly, individuals diagnosed with ADHD and their family members a chance to share their experiences and listen to other participants’ stories. The inclusion of diagnosed individuals and their caregivers in the Core Sets development has purposely been designed by the WHO and ICF Research Branch to involve a wide range of stakeholders. The four studies will together provide the basis for the ICF consensus conference, in which the first version of ICF(-CY) Core Sets for ADHD will be determined. The results of the first two preparatory studies, namely the comprehensive scoping review and expert survey, have already been published in previous issues of this journal [13, 35]. The remaining preparatory study of the ICF(-CY) Core Sets for ADHD project is a clinical cross-sectional investigation, with the objective to capture functional disability and strength in actual patients in naturalistic clinical settings. For the clinical study, participants will be recruited from clinics all over the world, thus making it possible to capture aspects of functioning and disability that might have been overlooked in this or previous studies or are specific to clinical environments. Once the first version of the ICF(-CY) Core Sets for ADHD has been defined, meaningful tools (e.g. diagnostic instruments, observation schedules, interviews) can be derived, psychometrically evaluated and used by stakeholders in different settings (e.g. research, clinical practice, health care administration and policy makers). Their implementation can aid to assess functioning and disability in individuals with ADHD, tailor treatment plans, follow-up intervention effects, and calculate related treatment resources.

## Electronic supplementary material

Below is the link to the electronic supplementary material.

Supplementary material 1 (DOCX 34 kb)
