# Supplementary material for: An international qualitative study of ability and disability in ADHD using the WHO-ICF framework
Source: Eur Child Adolesc Psychiatry. 2017 Mar 28;26(10):1219–31. doi: 10.1007/s00787-017-0983-1 (PMC5610225; doi:10.1007/s00787-017-0983-1)
Supplement: Supplementary file 1 — Supplementary material 1 (DOCX 34 kb) [file 787_2017_983_MOESM1_ESM.docx]

**Appendix 1.**


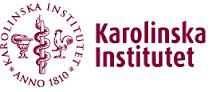

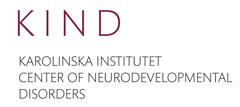


**Qualitative study on functioning and disability in ADHD –client and caregiver perspective**

**1. How does your ADHD affect the way your body and your mind works?** *(body functions)*

**2. In which parts of your body does your ADHD give you problems?** *(body structures)*

**3. How does your ADHD affect the things you can and cannot do you in your everyday life?** *(activities and participation)*

**4. What and/or who in the environment where you live and work/go to school make everyday life with ADHD difficult for you?** *(environmental factors -barriers)*

**5. What and/or who in the environment where you live and work/go to school is helpful and supportive in your everyday life with ADHD?** *(environmental factors –facilitators)*

**6. When you think about yourself and the person you are, what helps you to handle your everyday life with ADHD?** *(personal factors)*

**7. What can be the positive sides of living with ADHD?**
